# The emerging roles of MARCH8 in viral infections: A double-edged Sword

**DOI:** 10.1371/journal.ppat.1011619

**Published:** 2023-09-14

**Authors:** Changqing Yu, Qiang Liu, Zhuo Zhao, Jingbo Zhai, Mengzhou Xue, Yan-Dong Tang, Chengbao Wang, Chunfu Zheng

**Affiliations:** 1 Engineering Center of Agricultural Biosafety Assessment and Biotechnology, School of Advanced Agricultural Sciences, Yibin Vocational and Technical College, Yibin, People’s Republic of China; 2 Nanchong Key Laboratory of Disease Prevention, Control and Detection in Livestock and Poultry, Nanchong Vocational and Technical College, Nanchong, People’s Republic of China; 3 Beijing Centrebio Biological Corporation Limited, Beijing, People’s Republic of China; 4 Key Laboratory of Zoonose Prevention and Control at Universities of Inner Mongolia Autonomous Region, Medical College, Inner Mongolia Minzu University, Tongliao, People’s Republic of China; 5 Department of Cerebrovascular Diseases, The Second Affiliated Hospital of Zhengzhou University, 2 Jingba Road, Zhengzhou, People’s Republic of China; 6 State Key Laboratory for Animal Disease Control and Prevention, Harbin Veterinary Research Institute, Chinese Academy of Agricultural Sciences, Harbin, People’s Republic of China; 7 College of Veterinary Medicine, Northwest Agriculture and Forestry University, Xianyang, People’s Republic of China; 8 Department of Microbiology, Immunology & Infection Diseases, University of Calgary, Calgary, Canada; Fred Hutchinson Cancer Center, UNITED STATES

## Abstract

The host cell membrane-associated RING-CH 8 protein (MARCH8), a member of the E3 ubiquitin ligase family, regulates intracellular turnover of many transmembrane proteins and shows potent antiviral activities. Generally, 2 antiviral modes are performed by MARCH8. On the one hand, MARCH8 catalyzes viral envelope glycoproteins (VEGs) ubiquitination and thus leads to their intracellular degradation, which is the cytoplasmic tail (CT)-dependent (CTD) mode. On the other hand, MARCH8 traps VEGs at some intracellular compartments (such as the *trans*-Golgi network, TGN) but without inducing their degradation, which is the cytoplasmic tail-independent (CTI) mode, by which MARCH8 hijacks furin, a cellular proprotein convertase, to block VEGs cleavage. In addition, the MARCH8 C-terminal tyrosine-based motif (TBM) ^222^YxxL^225^ also plays a key role in its CTI antiviral effects. In contrast to its antiviral potency, MARCH8 is occasionally hijacked by some viruses and bacteria to enhance their invasion, indicating a duplex role of MARCH8 in host pathogenic infections. This review summarizes MARCH8’s antiviral roles and how viruses evade its restriction, shedding light on novel antiviral therapeutic avenues.

## Introduction

At least 11 members have been identified in the MARCH family, most containing 2 or more transmembrane domains (TMs), except for MARCH7 and MARCH10 without putative TMs [1]. Generally, the cytoplasmic C4HC3-type RING (Really Interesting New Gene)-finger domain (RING-CH finger) is located in MARCH proteins’ N-terminus, responsible for their E3 ubiquitin ligase activity. A tyrosine-based motif (TBM) YXXФ, responsible for endocytosis, is found in MARCH family proteins on their N-terminus, middle region, or C-terminus [2]. In addition, PDZ-binding domains and G/AxxxG/A motifs are also reported in MARCH proteins, which mediate protein–protein interactions and oligomerization [3,4]. MARCH proteins are generally located at the plasma membrane (PM) and some intracellular compartments, such as the endoplasmic reticulum (ER), the *trans*-Golgi network (TGN), and the mitochondrial membrane, or in the cytoplasm [3]. The MARCH family proteins are widely involved in a series of intracellular biological events regulation, including cell surface transmembrane proteins down-regulation [5], innate immunity signal transduction [1], and autophagy pathways regulation [6,7].

**Fig 1:**
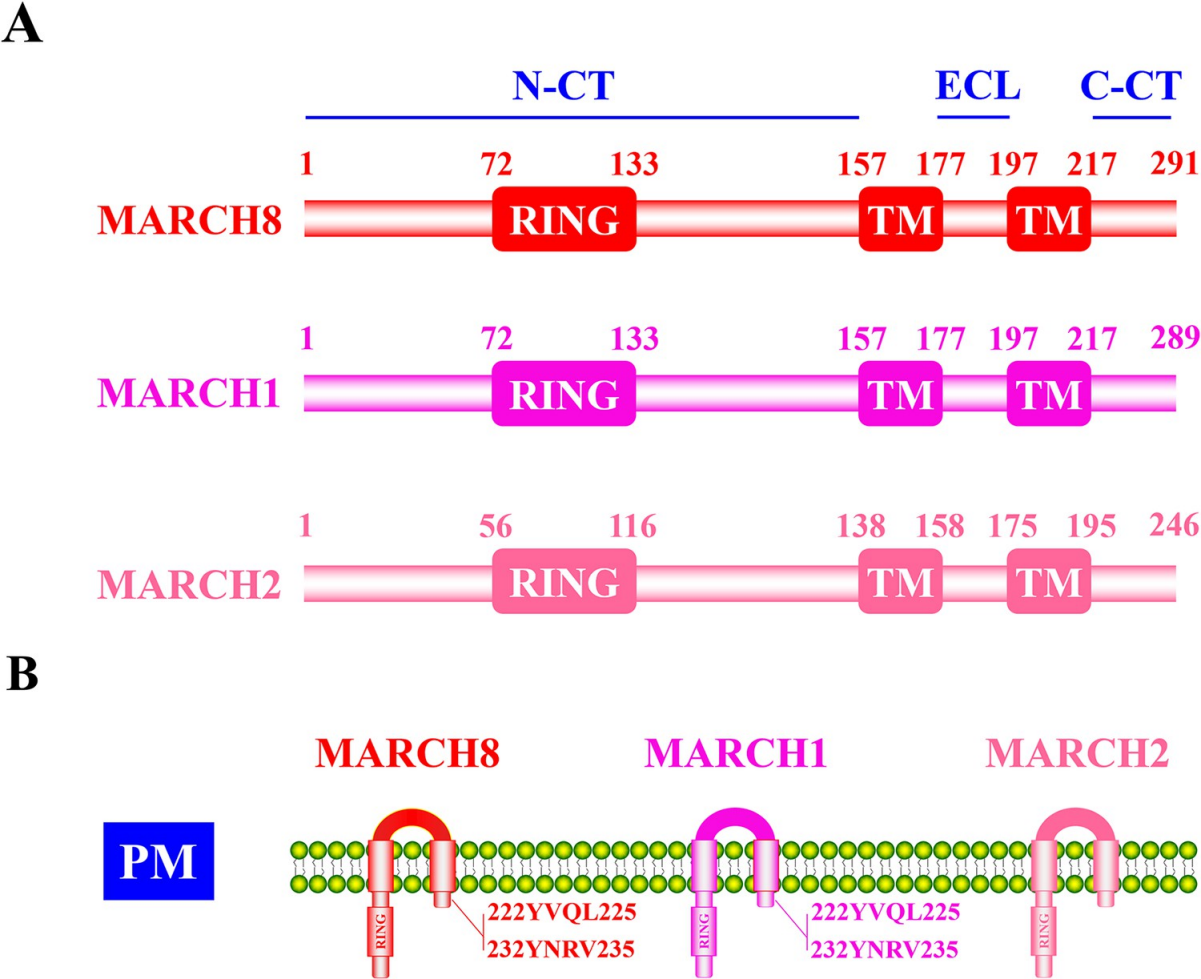
Schematic structure of human MARCH1, MARCH2, and MARCH8. (A) Primary structure of human MARCH1, MARCH2, and MARCH8. The human MARCH1, MARCH2, and MARCH8 contained an N-terminus RING domain, 2 TMs, an extracellular loop, and a C-terminus cytoplasmic tail. (B) Membrane-associated structure of human MARCH1, MARCH2, and MARCH8. Two TBMs, ^222^YVQL^225^ and ^232^YNRV^235^, are located in the C-terminus of MARCH1 and MARCH8. C-CT, C-terminus cytoplasmic tail; ECL, extracellular loop; N-CT, N-terminus cytoplasmic tail; PM, plasma membrane; RING, E3 ubiquitin ligase domain; TBM, tyrosine-based motif; TM, transmembrane domain.

MARCH8, the first identified MARCH family protein, was originally named cellular modulator of immune recognition (c-MIR; also known as MARCHF8) because of its functional and structural homolog to Kaposi’s sarcoma-associated herpesvirus (KSHV) MIR-1 and MIR-2 (also termed K3 and K5, the membrane-associated E3 ligase) [8]. Structurally, human MARCH8 contains a typical N-terminus RING domain, 2 TMs, and 2 C-terminus TBMs (Fig 1). MARCH8 is located at intracellular compartments, such as the early and late endosomes, or the cell surface [3,5], and it has been recently characterized to trap viral glycoproteins at the TGN [9,10]. Among human MARCH family proteins, MARCH1 shows a high sequence and structure homology to MARCH8 [11] (Fig 1), which is supposed to execute redundant intracellular immunoregulatory role, such as down-regulation of CD86 and transferrin receptor (TfR) [12]. Oligomerization is found among these MARCH proteins. For example, MARCH1, MARCH8, and MARCH9 could form homodimers or heterodimers, which are supposed to influence their autoubiquitination and intracellular turnover [13]. In addition, distinct isoforms of MARCH proteins display different activities [3]. Two isoforms of MARCH1 are indicated to show significant differences in their anti-influenza A virus (IAV) potency [14]. MARCH2 also shares a structural similarity with MARCH8 and is involved in signal transduction [1,15] (Fig 1). MARCH8 participates in the steady-state regulation of many membrane-associated proteins, including major histocompatibility complex II (MHC II) [16,17], CD86 [18], CD98 [19], TfR [20], TNF-related apoptosis-inducing ligand receptor 1/2 (TRAIL-R1/2) [21,22], programmed death ligand 1 (PD-L1) [23], and interleukin-1 receptor accessory protein (IL-1RAP) [24]. Besides, MARCH8 is also involved in signal transduction regulation of antiviral innate immunity responses. For example, MARCH8 is recruited by tetherin (also termed bone marrow stromal antigen 2, BST-2) to promote mitochondrial antiviral signaling protein (MAVS) degradation through nuclear dot protein 52 kDa (NDP52)-mediated selective autophagy [6,25].

Importantly and interestingly, MARCH proteins are discovered to execute antiviral functions [26]. Tada and colleagues first reported that MARCH8 inhibited HIV-1 infection by reducing its envelope glycoprotein incorporation into virions [27]. Subsequently, many studies reported that MARCH8 restricted infection by a variety of viruses [28], and the antiviral members of MARCH proteins are extended to include MARCH1 and MARCH2 of different mammalian origins [14,29–32]. The viruses targeted by these MARCH proteins now include HIV-1 [27,30,33], Severe Acute Respiratory Syndrome Coronavirus 2 (SARS-CoV-2) [31,33,34], ebola virus (EBOV) [10,33], IAV [14,35,36], and many other highly pathogenic zoonotic viruses [31,34]. Generally, MARCH8 employs 2 antiviral modes in suppressing infection by these enveloped viruses, i.e., the CTD and CTI modes mentioned above [9,10,33]. This mini-review will summarize MARCH8’s current antiviral potency under these modes and illustrate its immunoregulatory roles, intending to provide insights for later antiviral studies.

## The CTD antiviral mode

Under the CTD mode, MARCH8 generally ubiquitinates the viral glycoproteins via recognition of their cytoplasmic tails, mediating their endocytosis and targeting them for lysosome degradation, which was reported by many studies [27,33–35,37].

### Vesicular stomatitis virus G-glycoprotein (VSV-G)

**Fig 2:**
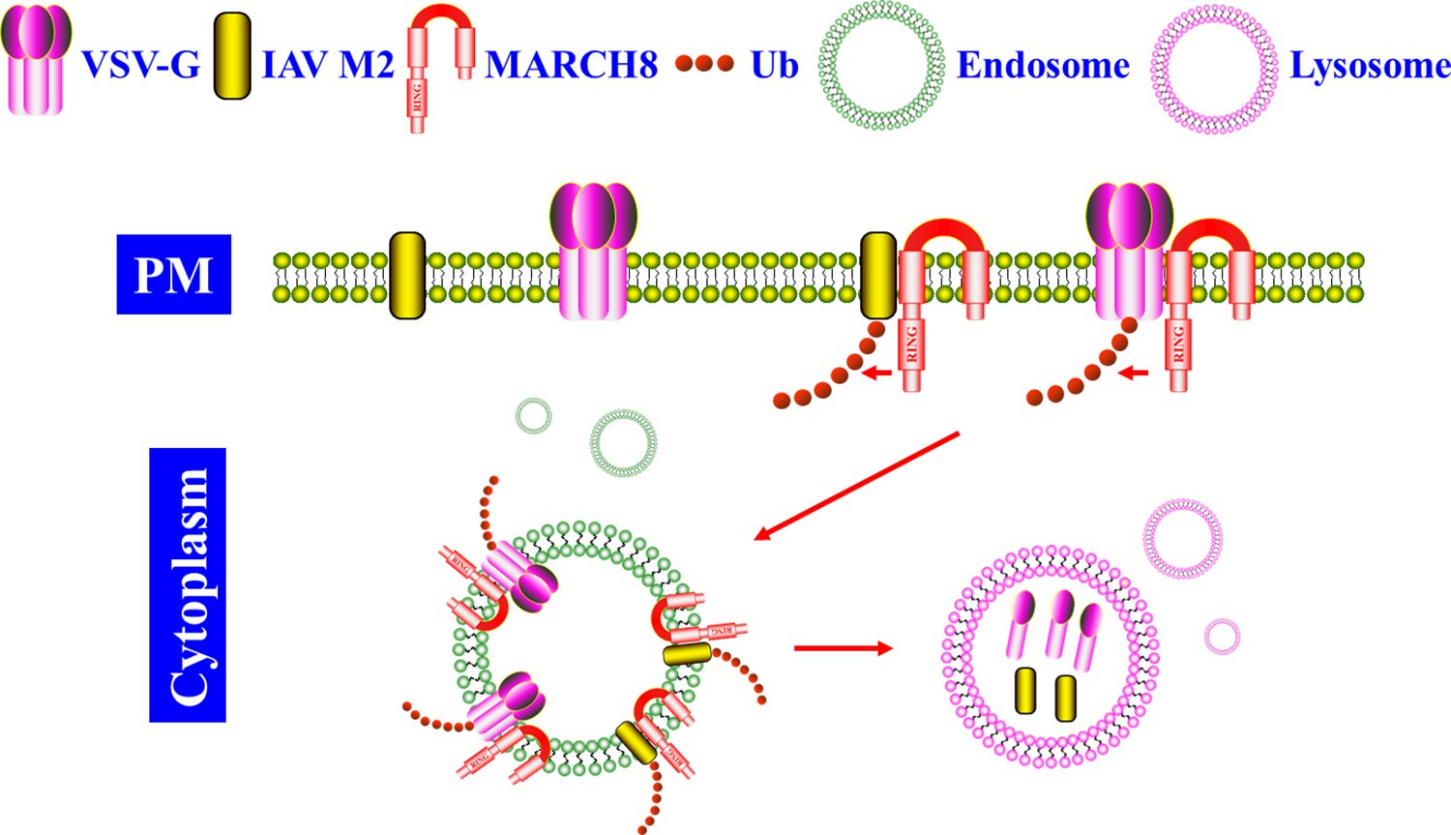
The MARCH8 CTD antiviral mode. MARCH8 generally recognized viral proteins’ cytoplasmic tail lysine residues and catalyzed their polyubiquitination, which thus induced these viral proteins’ translocation from the cell PM and entering into the lysosome pathway for degradation. Other viral glycoproteins targeted by MARCH8 via the CTD mode shared a similar degradation route to VSV-G and IAV H1N1 M2. CTD, cytoplasmic tail-dependent; PM, plasma membrane; Ub, ubiquitin; VSV-G, vesicular stomatitis virus G-glycoprotein.

Initially, Tada and colleagues discovered that VSV-G pseudotyped lentiviral cores from MARCH8-expressing cells were significantly less infectious [27]. It was found that MARCH8 induced VSV-G intracellular degradation (Fig 2), whereas lysosome inhibitors helped restore its expression [10,27]. VSV-G cytoplasmic tail (CT) contained several lysine residues targeted for ubiquitination by MARCH8. Replacing these lysine residues with arginine residues or a CT truncation enabled VSV-G to resist MARCH8-imposed restriction [9,33]. These studies demonstrated a CTD antiviral mode of MARCH8 in restricting VSV-G.

### Murine leukemia virus (MLV) envelope glycoprotein (Env)

Originally, human MARCH8 was found to inhibit MLV Env-mediated pseudovirion infection [27]. Later, Umthong and colleagues explored the antiviral activities of mouse MARCH family proteins, including MARCH1, MARCH2, MARCH3, and MARCH8 [31]. Their work demonstrated that mouse MARCH1/8 effectively suppressed MLV infection by targeting MLV Envs (gp70 and p15E) to the lysosome pathway given that lysosome inhibitors rescued MLV gp70/p15E intracellular expression. Importantly, p15E CT truncation rendered gp70/p15E resistant to mouse MARCH1/8 induced intracellular degradation, suggesting mouse MARCH1/8 antagonized MLV gp70/p15E via the CTD antiviral mode [31]. Mouse MARCH1/8 also abrogated mouse mammary tumor virus (MMTV) gp36 intracellular expression and virion incorporation, probably through the CTD antiviral mode [31]. Critically, the MLV glycoGag was reported to counteract host serine incorporator 5 (SERINC5) [38–40] and interferon-induced transmembrane protein 3 (IFITM3) [41], which thus enhanced its infectivity. Therefore, further studies should investigate whether MLV glycoGag could neutralize mouse MARCH1/8-imposed restriction.

### IAV matrix 2 (M2) protein

Current reports indicate that MARCH8 generally targets viral envelope spike glycoproteins under the CTD antiviral mode. However, the other viral proteins could also be restricted by MARCH8. Recently, Liu and colleagues discovered MARCH8 profoundly suppressed IAV infection in vitro and in mice [35]. Further dissection uncovered MARCH8 targeted IAV H1N1 M2 protein for lysosome degradation (Fig 2), thereby preventing virions release from the cell surface. K63-linked polyubiquitination at M2 CT K78 was critical for MARCH8 recognition and the subsequent intracellular degradation. Interestingly, via K78/K79 variation into non-lysine residues in M2, the IAV H1N1 became partially resistant to MARCH8-imposed replication restriction and, thus, more virulent [35]. Other than M2, the IAV hemagglutinin (HA) glycoprotein is also targeted by human MARCH8 [10,31,36], which we will discuss later.

### Spring viremia of carp virus G-glycoprotein (SVCV-G)

In a recent study, Li and colleagues found that heat shock cognate protein 70 (HSC70) negatively regulated SVCV replication [37]. Their research identified that HSC70 interacted with SVCV-G and promoted its degradation in a dose-dependent manner. The lysosome inhibitor could restore SVCV-G intracellular expression. In-depth work uncovered zebrafish MARCH8 (z-MARCH8) interacted with both HSC70 and SVCV-G and promoted SVCV-G lysosome degradation, thus inhibiting SVCV replication. In contrast, SVCV-G CT truncation abrogated z-MARCH8-induced degradation. The SVCV-G CT harbored 2 lysines, both crucial for z-MARCH8 recognition because only substituting one of the 2 lysines in SVCV-G CT made it still sensitive to z-MARCH8-induced degradation [37]. These lines of evidence proved that MARCH8 reduced SVCV replication via the CTD antiviral mode.

### Raby virus G-glycoprotein (RABV-G) and other viral envelope glycoproteins (VEGs)

Besides the viruses mentioned above, MARCH8 also recognized CT lysine residues of many other VEGs and diminished their cellular expression, including RABV-G, lymphocytic choriomeningitis virus glycoprotein (LCMV-GP), SARS-CoV and SARS-CoV-2 spike (S) glycoprotein, Chikungunya virus (CHIKV), and Ross River virus (RRV) envelope glycoprotein (Env) [31,34]. MARCH8 lowered the infectivity of virions pseudotyped by these VEGs in a dose-dependent manner, whereas the lysosome inhibitor relieved the infection inhibition by MARCH8. In addition, the CT K-to-R mutations made these VEGs resistant to MARCH8-imposed restriction [34], demonstrating MARCH8 impaired their function in a CTD mode. Notably, MARCH8-stably expressing cells inhibited infection of the RABV strain CVS-26 [34], arguing that MARCH8 may also potently restrict fully replication-competent viruses.

### Modulation of autophagy pathways and innate immunity signaling

Besides regulating viral transmembrane glycoproteins, MARCH8 was also found to target cytoplasmic viral proteins for degradation. This antiviral mode of MARCH8 is similar to the CTD pattern, which also depends on its E3 ligase activity.

**Fig 3:**
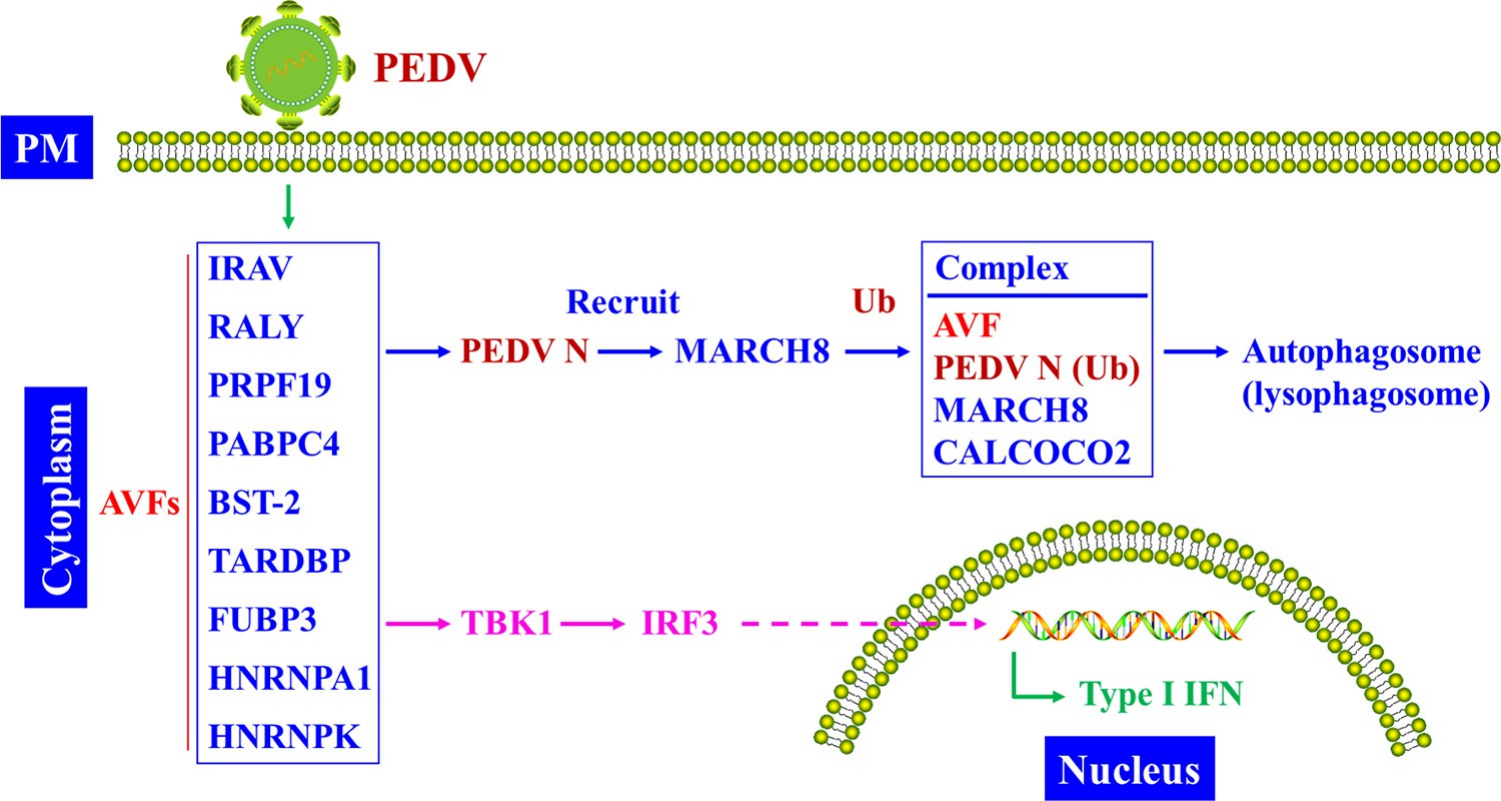
MARCH8 regulated viral protein autophagy pathway. After entry into host cells, the PEDV N protein was recognized and hijacked by a series of host proteins, such as IRAV, PRPF19, and HNRNPK. These host antiviral proteins recruited MARCH8 to ubiquitinate PEDV N protein and combined with CALCOCO2 to form a cellular complex directed to autophagosome (lysophagosome)/proteasome for degradation. In addition, the host proteins, including TARDBP, FUBP3, HNRNPA1, and HNRNPK, also activated the production of cellular type-I IFN, which enhanced the host cell antiviral immunity responses. AVF, antiviral factor; IRF3, interferon regulatory factor 3; PEDV, porcine epidemic diarrhea virus; PM, plasma membrane; TBK1, TANK binding kinase 1; Ub, ubiquitination.

Previously, Roy and colleagues found that MARCH8 could ubiquitinate BST-2 and promote its lysosome degradation [42]. Recent work indicated that BST-2 also hijacked MARCH8 to initiate the cellular autophagy signaling pathway. Jin and colleagues discovered BST-2 specifically suppressed RNA virus-induced type-I interferon (IFN) signaling by targeting MAVS [6]. BST-2 recruited MARCH8 to ubiquitinate MAVS and induce it into the NDP52/CALCOCO2-mediated selective autophagy pathway. Specifically, MARCH8 catalyzed K27-linked ubiquitination on MAVS K7 and thus led to its autophagic degradation. Interestingly, this pathway is recently confirmed to be utilized by BST-2 to counteract porcine epidemic diarrhea virus (PEDV) infection [7]. Like MAVS ubiquitination, BST-2 hijacked MARCH8 to ubiquitinate PEDV nucleocapsid (N) protein and induced its entry into the CALCOCO2-directed autophagy pathway, which thus suppressed PEDV infection [7]. Besides BST-2, many other host factors were also identified to utilize MARCH8 to block PEDV infection. Up-to-date, these antiviral factors include IFN-regulated antiviral protein (IRAV) [43], Poly(A)-binding protein 4 (PABPC4) [44], trans-active response DNA-binding protein (TARDBP) [45], far upstream element-binding protein 3 (FUBP3) [46], RNA-binding protein (RALY, also known as hnRNPCL2) [47], heterogeneous nuclear ribonucleoprotein K (hnRNP K) [48], pre-mRNA processing factor 19 (PRPF19) [49], and heterogeneous nuclear ribonucleoprotein A1 (hnRNPA1) [50] (Fig 3). Notably, PABPC4 could recruit MARCH8 and induce autophagosome degradation of the N proteins of 4 coronavirus (CoV) genera [44], including *Alphacoronavirus*, *Betacoronavirus* (such as SARS-CoV, SARS-CoV-2, and Middle East respiratory syndrome (MERS)-CoV), *Gammacoronavirus*, and *Deltacoronavirus*, demonstrating MARCH8-mediated crucial and extensive role in anti-CoV infection. Intriguingly, the 2AB protein of senecavirus A (SVA), an emerging porcine virus, promoted MARCH8 degradation via binding to LC3 or MAVS [51] and thus antagonized cellular selective autophagy and type-I IFN production, which was beneficial for virus replication, arguing that the virus needs to evolve a mechanism to counteract MARCH8-exerted restriction. Notably, SVA infection of PK-15 cells up-regulated MARCH8 mRNA transcription [51], implying SVA 2AB inhibited MARCH8 accumulation posttranscriptionally.

Intriguingly, Yang and colleagues recently discovered that MARCH8 negatively regulated the innate immunity signaling pathway [52]. They found that MARCH8 catalyzed the K63-linked polyubiquitination of cyclic GMP-AMP synthase (cGAS), which did not induce its degradation but impeded its binding to DNA and thus attenuated the downstream innate immunity responses. In concordance with this, *March8*-deficient mice were less susceptible to HSV-1 infection than their wild-type littermates and displayed delayed death [52], implying the cGAS activity repression by MARCH8 was relieved, and the host innate immunity responses were thus enhanced to defend against viral infection. Among human MARCH family members, besides MARCH8, MARCH1, MARCH2, and MARCH5 also regulated cellular innate immunity signaling pathways [1].

## The CTI antiviral mode

Unlike the CTD mode, MARCH8 does not induce VEGs degradation but inhibits their intracellular maturation and sequesters them in some intracellular compartments under the CTI mode [28], which has already been reported by many antiviral studies [9,10,27,33].

### HIV-1 envelope glycoprotein (Env)

**Fig 4:**
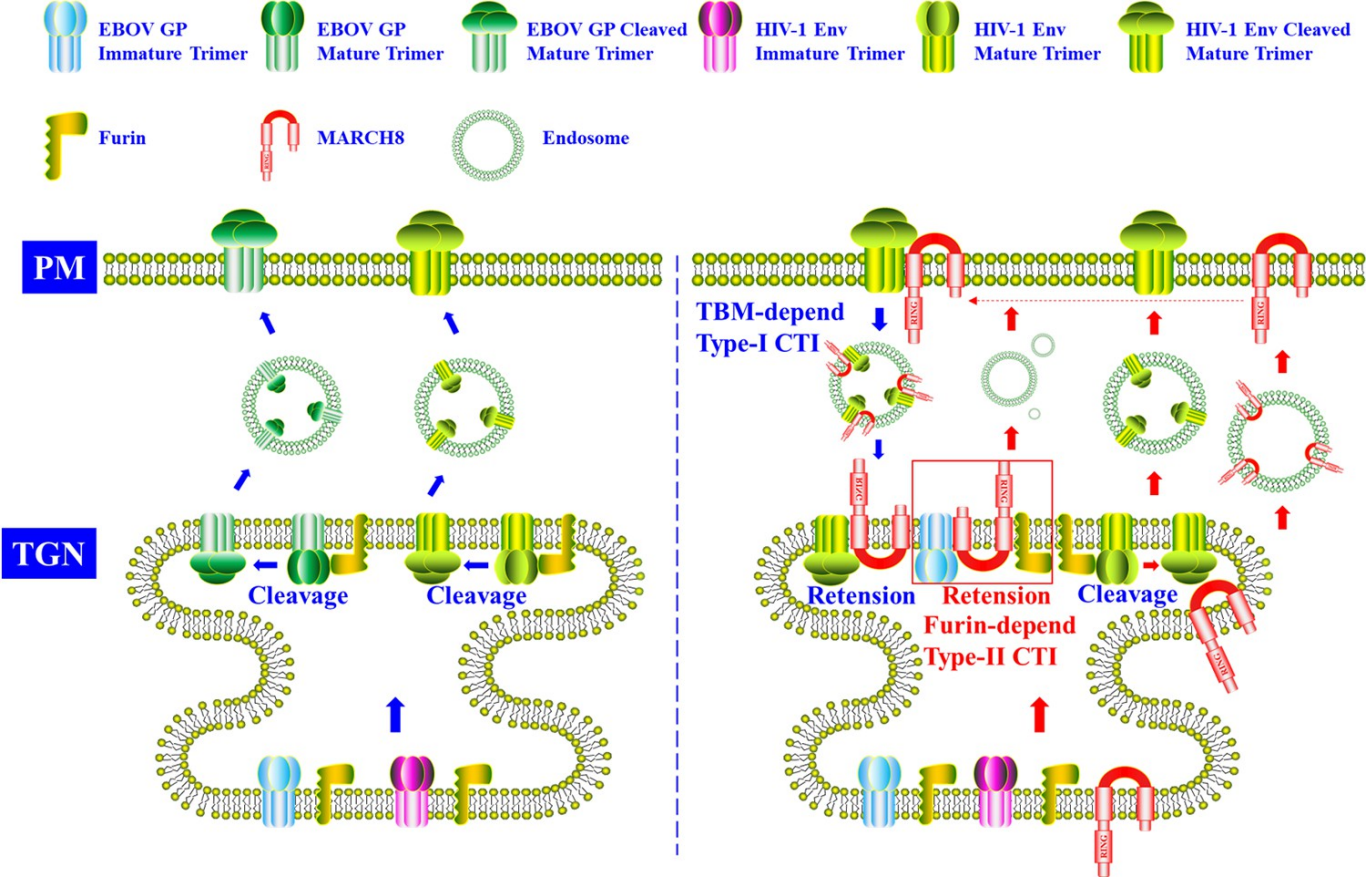
The MARCH8 CTI antiviral mode. This figure is adapted from Fig 3 of a previous publication [28]. MARCH8 employed the TBM-dependent (type-I) and furin-dependent (type-II) CTI modes to restrict HIV-1 Env and EBOV GP activities, respectively. Under the type-I CTI mode, HIV-1 Env was down-regulated by MARCH8 from the cell PM, where it was translocated to and trapped at the TGN apparatus. However, under the type-II CTI mode, EBOV GP was retained by MARCH8 at the TGN apparatus, where it was prevented from anterograde transport to the cell PM. Simultaneously, the EBOV GP cellular cleavage and glycosylation maturation were also blocked by MARCH8 at the TGN. Human MARCH8 probably restricts SARS-CoV-2 S and IAV H5N1 HA through the type-II CTI antiviral mode. CTI, cytoplasmic tail-independent; HA, hemagglutinin; IAV, influenza A virus; PM, plasma membrane; SARS-CoV-2, Severe Acute Respiratory Syndrome Coronavirus 2; TBM, tyrosine-based motif; TGN, *trans*-Golgi network.

It has been reported that MARCH8 restricted VSV-G and HIV-1 Env function via different mechanisms. MARCH8 employed the CTD mode to inactivate VSV-G while it engaged the CTI machinery to subvert HIV-1 Env [9,10,33]. Under the CTI mode, MARCH8 did not target HIV-1 Env for intracellular degradation but down-regulated it from the PM and sequestered it in intracellular compartments [27,33] (Fig 4), such as the TGN [9], which thus reduced the infectivity of nascent virions [9,27,33].

Recent reports indicated that the MARCH8 CTI antiviral mode could be divided into at least 2 categories. The first could be defined as TBM-dependent (type-I) based on Zhang and colleagues’ work [9]. They discovered that the HIV-1 Env CT K-to-R variant was still sensitive to MARCH8 restriction, whereas the VSV-G was not, indicating that MARCH8 inactivated HIV-1 Env independent of its CT. As introduced above, the MARCH8 C-terminus CT harbors 2 TBMs, i.e., the YXXФ sequence (X and Ф stand for any amino acid and hydrophobic residues, respectively), which is involved in clathrin-dependent endocytosis. Zhang and colleagues identified that the MARCH8 ^222^YxxL^225^ was critical for its CTI antiviral potency [9,34]. Their work indicated the MARCH8 ^222^AxxL^225^ mutant failed to inhibit HIV-1 infection but potently restricted VSV-G/LCMV-GP pseudotyped virions infectivity [9,34], pointing out that this type of MARCH8 anti-HIV-1 CTI mode was TBM-dependent. Following the phenotype by Zhang and colleagues, Lun and colleagues found full-length HIV-1 Env or a CT deletion was restricted by MARCH8, while the tailless VSV-G was resistant to MARCH8 [33]. Besides, they also identified a cleavage deficiency of HIV-1 Env under MARCH8 inhibition. Consistent with this work, Yu and colleagues reported that MARCH8 blocked HIV-1 Env cleavage via binding to the cellular proprotein convertase furin [10]. In their study, MARCH8 was found not to induce HIV-1 Env intracellular degradation but instead blocked the HIV-1 Env cleavage via formation of a cellular MARCH8-furin-HIV-1 Env complex [10]. MARCH8 CTI antiviral mode of this type can be defined as the second furin-dependent (type-II). These studies together demonstrated that MARCH8 suppressed HIV-1 Env cleavage by targeting furin and retained it intracellularly [9,10,33].

It is yet to be determined whether the MARCH8 ^222^AxxL^225^ mutant fails to bind furin and thus leads to its incapacity of retaining HIV-1 Env intracellularly. If this were the case, the 2 CTI modes mentioned above should act in concert. Future investigations are necessary to explore this option.

### EBOV glycoprotein (GP)

Mature EBOV GP contains a polybasic site and could be cleaved by endogenous furin into 2 subunits, GP_1_ and GP_2_, which are then relinked and transported to the PM as spikes when virions bud from cells [53]. In a recent study, Yu and colleagues discovered that MARCH8 inhibited EBOV GP maturation via the type-II CTI antiviral mode [10]. As in the case of HIV-1, it was dissected that MARCH8 did not promote EBOV GP intracellular degradation but suppressed its proteolytic cleavage and glycosylation maturation (Fig 4). Further analysis indicated MARCH8 interacted with EBOV GP and furin and retained them at the TGN, thus blocking EBOV GP anterograde transportation to the PM [10]. Consistent with this study, Lun and colleagues observed MARCH8 impaired EBOV GP intracellular cleavage, dramatically reducing cellular and virion-associated GP_1_ and GP_2_ [33]. Importantly, the tailless GP or GP CT K-to-A variant was still sensitive to MARCH8 restriction, ruling out the CTD mode and verifying the CTI antiviral mode engaged by MARCH8 towards EBOV GP [33]. These studies demonstrated MARCH8 trapped EBOV GP in intracellular compartments. It is speculated that MARCH8 degrades some critical cellular trafficking proteins involved in EBOV GP translocation. However, the precise underlying mechanism needs to be further explored.

Besides MARCH8, many host antiviral factors employed the type-II CTI mode to block VEGs cleavage [28,54]. The guanylate-binding protein 2/5 (GBP2/5) and α-soluble N-ethylmaleimide sensitive fusion (NSF) attachment protein (α-SNAP) inhibited furin-dependent cleavage of HIV-1 Env [55,56], SARS-CoV-2 S [57], IAV H5N1 HA [56], and EBOV GP [57]. MARCH8 and α-SNAP recognized the furin P domain and blocked EBOV GP cleavage [32,57]. Thus, it would be interesting to investigate whether they have intracellular interactions. In addition, bovine and murine MARCH8 also blocked EBOV GP cleavage [10], implying a conserved CTI antiviral mode across mammalian species. Furthermore, whether the type-I CTI antiviral mode is critical for MARCH8 restriction of EBOV GP should be examined in later work.

### SARS-CoV-2 S

Several studies demonstrated that MARCH8 restricted SARS-CoV-2 S activity through distinct modulation modes. Lun and colleagues discovered MARCH8 inhibited the pseudovirus infectivity of SARS-CoV-2 S enveloped HIV-1 cores [33]. Further analysis indicated MARCH8 blocked SARS-CoV-2 S proteolytic processing and trapped it in an intracellular compartment. In addition, the S-cleavage product S_2_ was demonstrated to be less glycosylated, implying the S glycoprotein maturation was suppressed. Importantly, the S CT truncation or its CT K-to-R variant was still sensitive to MARCH8-imposed restriction [33]. These lines of evidence indicated MARCH8 impaired SARS-CoV-2 S function via the CTI but not the CTD antiviral mode. However, as described above, Zhang and colleagues showed a CTD antiviral mechanism [34]. They found bafilomycin A1, a lysosome inhibitor, helped restore the SARS-CoV-2 S-pseudotyped virions infectivity, and the S_2_ CT K-to-R variation made it insensitive to MARCH8 restriction, implying the MARCH8 CTD antiviral effects on SARS-CoV-2 S. Intriguingly, less S_2_ glycosylation modification was also observed in Zhang and colleagues’ work [34]. Similar to this finding, another report by Umthong and colleagues demonstrated human MARCH8 reduced the intracellular expression of SARS-CoV-2 S and highly glycosylated M proteins [31], arguing a CTD antiviral potency of MARCH8. According to these results, different MARCH8 anti-SARS-CoV-2 S modes are described. The inconsistency of these results may arise from the differential operating systems these VEGs employed. Or else, MARCH8 can utilize the CTI and CTD antiviral modes targeting the same viral protein but only prefers 1 mode under specific physiological conditions. In any case, more in-depth work is needed to address these questions.

### IAV HA

In addition to the M2 protein, as discussed above, MARCH8 also targeted IAV HA to suppress its infection. Several groups described distinctive regulation modes of MARCH8 on IAV HA. MARCH8 was indicated to reduce IAV H1N1 HA intracellular expression, implying a CTD antiviral mode [31]. However, for the highly pathogenic IAV H5N1 HA, which contained a furin-cleavage site, MARCH8 was shown to block its glycosylation maturation and cleavage into the HA_1_ and HA_2_ subunits [10], indicating a CTI antiviral mode. Intriguingly, Villalón-Letelier and colleagues’ work discovered that MARCH8 neither induced HA/NA (neuraminidase)/M2 intracellular degradation and cell surface down-regulation nor inhibited the HA proteolytic cleavage but instead reduced the HA/M1/M2 virion incorporation [36], arguing MARCH8 influenced IAV assembly or budding and suggesting that MARCH8 employed a novel pattern distinguished from the known CTD/CTI antiviral modes, which needs further elucidation. Their work also identified that the ^222^YxxL^225^ motif was crucial for MARCH8 anti-IAV infection [36]. This motif is thought to interact with some critical proteins responsible for IAV assembly and release.

MARCH8 was reported to interact with furin and EBOV GP and trapped them intracellularly [10], indicating MARCH8 utilized furin as the bridge to retain EBOV GP. The IAV H5N1 HA also harbors a furin-recognition site. Thus, more studies are needed to investigate whether MARCH8 could retain IAV H5N1 HA intracellularly via the furin bridge.

## Supportive role in pathogenic infections

In contrast to its antiviral potency, MARCH8 has been reported to be utilized by viruses or bacteria to enhance their intracellular infection. Hepatitis C virus (HCV) nonstructural protein 2 (NS2) hijacked MARCH8 to catalyze its K63-linked ubiquitination and thus recruited the host endosomal sorting complex required for transport (ESCRT) machinery to promote viral assembly and envelopment [58,59]. Notably, Kumar and colleagues discovered that other host E3 ligases, other than MARCH8, were also involved in this process [59]. In addition to HCV, MARCH8 was also beneficial for Zika virus (ZIKV) and dengue virus (DENV) infection [59], illustrating viruses hijacked cellular E3 ligases to enhance their infection may be a commonly utilized mode.

Coincidence with this work, a recent study showed that SVCV infection of zebrafish tissues/liver cells significantly up-regulated z-MARCH8 transcription [60]. The z-MARCH8 then targeted cellular TANK-binding kinase 1 (TBK1) and mediator of IRF3 activator (MITA) for intracellular degradation, which down-regulated IFN responses and thereby facilitated SVCV and grass carp reovirus (GCRV) infection, consistent with the negative regulation of MARCH8 on innate immunity signaling, as discussed above [52].

Similar to MARCH8 engagement by some viruses, the bacteria were also found to utilize the negative immunoregulatory role of MARCH8 to evade immunosurveillance and thus aid their cellular infection. Bayer-Santos and colleagues uncovered that the *Salmonella* effector protein SteD interacted with both MARCH8 and the mature MHC II (mMHC II), promoting MARCH8-dependent ubiquitination of the β-chain CT of mMHC II, which thereby reduced the mMHC II cell surface abundance, inhibited antigen presentation, and consequently suppressed CD4^**+**^ T cell activation [61]. These studies illustrated how some microbial pathogens, e.g., HCV, SVCV, and *Salmonella*, could hijack MARCH8 to favor their intracellular infections, indicating a complex coevolutionary counteraction between the pathogens and host cells.

## Other antiviral members

The MARCH family now contains 11 members. Based on current reports, besides human MARCH8, other mammalian MARCH8 molecules or other MARCH family members were also found to display antiviral activities through the CTD and CTI modes. MARCH8 from mice and cattle inhibited MLV and EBOV GP-pseudotyped virus infection via either the CTD or the CTI mode [10,31]. Similarly, the mouse MARCH1/8 but not MARCH2 significantly inhibited MLV infection via the CTD antiviral mode [31]. In addition, human MARCH1 and MARCH2 were indicated to suppress VSV-G/RABV-G/LCMV-GP/CHIKV Env/RRV Env-mediated pseudovirus infection [30,34] and reduced the intracellular levels of IAV H1N1 HA and highly glycosylated SARS-CoV-2 M protein [31], implying the CTD antiviral mode. In contrast, human MARCH1 and MARCH2 inhibited HIV-1 infection via the CTI antiviral mode [30]. Similar to MARCH8, MARCH1 also contained C-terminus TBMs (Fig 1). Whether the TBMs played important roles in MARCH1 antiviral immunity needs to be investigated. Human MARCH1 and MARCH2 were recently discovered to suppress EBOV GP intracellular cleavage via blocking furin activity [32], as human MARCH8 did [10], indicating a type-II CTI antiviral mode. These results demonstrated that MARCH family proteins shared a conserved inter and intraspecies antiviral potency and indicated a broad antiviral spectrum under the CTD and CTI modes.

## Concluding remarks

This review summarizes the antiviral potency of MARCH8 and its 2 close family members, MARCH1 and MARCH2. Generally, these MARCH proteins employed 2 typical modes in their antiviral function, i.e., the CTD and CTI. As the role in down-regulating cell surface immunity molecules [5], MARCH8 catalyzed the VEGs ubiquitination via the CTD mode and thus promoted their entry into the degradation pathways, diminishing the viral glycoprotein-dependent virions infectivity for diverse VEGs, including the VSV-G, MLV Env, SVCV-G, and RABV-G [27,31,33,34,37]. However, under the CTI antiviral mode, MARCH8 generally did not induce VEGs degradation but inhibited their biological activities, including glycosylation maturation and proteolytic cleavage. Importantly, the TBM ^222^YxxL^225^ played a key role in MARCH8 CTI antiviral mode [9,34]. The C-terminus CT Y222A variation of MARCH8 could not restrict HIV-1 Env/RABV-G/SARS-CoV S/SARS-CoV-2 S but maintained protein inhibition of VSV-G/LCMV-GP potently [9,34], indicating MARCH8 utilized distinct antiviral modes to restrict different clades of VEGs. Notably, MARCH8 harboring the ^222^AxxL^225^ mutation showed a low-level expression [36], probably thus reducing its antiviral potency. However, this phenotype was not observed in its anti-HIV-1 infection [9] and needs further clarification.

Particularly, under the CTI mode, MARCH8 could hijack cellular furin to block the cleavage and maturation of a group of VEGs, including HIV-1 Env [10,33], EBOV GP [10,33], IAV H5N1 HA [10], and probably SARS-CoV-2 S [33]. Furin cleaves many viral glycoproteins and cellular proproteins [54], arguing that MARCH8 probably inhibits a broad range of viral proteins and processing of cellular proteins other than those mentioned above. Indeed, many host antiviral factors, such as GBP2/5 [55,56], protease-activated receptor 1 (PAR1) [62–64], α-SNAP [57], and possibly lectin galactoside-binding soluble 3 binding protein (LGALS3BP/90K) [65,66] targeted furin to restrict infection by a group of viruses, demonstrating a conserved cellular type-II CTI antiviral mode.

Besides inhibiting their cleavage and maturation, MARCH8 sequestered HIV-1 Env, EBOV GP, and SARS-CoV-2 S at the TGN or some intracellular compartments [9,10,33]. Critically, beyond the CTD and CTI antiviral modes, MARCH8 was found neither to interfere with IAV H1N1 HA/M1/M2 intracellular expression nor their PM localization but reduced their assembly into virions [36]. Putatively, MARCH8 may ubiquitinate some crucial host trafficking machinery/assembly proteins and promote their translocation or degradation via recognition of their CT, thus indirectly blocking the viral glycoprotein transportation to the PM or assembly into virion particles. In any case, more in-depth studies are needed to dissect the underlying mechanisms.

MARCH8 ubiquitinated its substrates via the E3 ligase activity in the CTD antiviral mode. Thus, the E2 ubiquitin-conjugating enzymes (E2 enzymes) are involved in this process. Under the CTI antiviral mode, MARCH8 did not target the viral glycoproteins for degradation, indicating a lack of E2 enzyme assistance. However, a MARCH8 RING domain mutant (W114A, W114 is conserved among MARCH family and many other RING finger E3 ligases and responsible for E2 enzymes recruitment [24]), which loses the E3 ligase activity, abrogated its CTI antiviral effects [10,27,33]. Therefore, it can not rule out the possibility that some E2 enzymes are involved in MARCH8 CTI antiviral processes. Thus, it would be interesting to identify the E2 enzymes cooperating with MARCH8 in its CTD mode and explore whether the E2 enzymes participate in its CTI antiviral effects. Importantly, some E2 enzymes, such as UbcH2/UbcH5a [12] and UbcH5c [24], were demonstrated to be involved in the ubiquitination of MARCH8 itself or its substrate. These studies should provide clues for identifying E2 enzymes that cascade with MARCH8 in its CTD and possibly the CTI antiviral mode. In addition, current investigations mainly focus on viral glycoproteins lysosome degradation under the MARCH8 CTD antiviral mode, whereas fewer studies check whether these targeted viral glycoproteins follow the autophagy pathway. Therefore, whether MARCH8 or MARCH1/2 utilizes autophagy signaling pathways to clear the viral glycoproteins deserves further explanation.

Based on current reports, MARCH8 employs CTD and CTI modes to antagonize infection by the same virus. Investigators found that MARCH8 utilized the CTD and CTI modes to restrict HIV-1 Env, SARS-CoV-2 S, and IAV HA function [10,27,31,33,34]. The conclusions drawn by different groups mainly depended on their respective experimentation systems, most of which were performed in vitro. Thus, in vivo studies need to validate the elaborate antiviral mode of MARCH8 in the future.

Interestingly, current studies indicate MARCH8 also targets multiple viral proteins of one kind of virus. For example, MARCH8 induced IAV H1N1 M2 and HA degradation [31,35], blocked IAV H5N1 HA cleavage [10], and reduced IAV H1N1 HA/M1/M2 incorporation into virions [36]. Similarly, MARCH8 induced SARS-CoV-2 N/M/S intracellular degradation [31,34,44] and blocked the S glycoprotein cleavage and glycosylation maturation [33]. However, despite MARCH8’s potent activities against these vital viral proteins, not all viruses are indicated to evolve counteracting strategies. Overall, the virus-MARCH8 cellular contradiction can be classified into 3 cases:

(I) The virus does not evolve a viral gene to resist MARCH8. Tada and colleagues reported MARCH8 potently restricted HIV-1 infection in monocyte-derived macrophages (MDMs) and knockdown/knockout of MARCH8 enhanced HIV-1 replication in MDMs [27]. However, HIV-1 has not evolved a viral gene to antagonize MARCH8. As previously described, MARCH8 down-regulates cell surface MHC-II and some co-stimulatory molecules [5], which play pivotal roles in activating cellular T-cell responses [2]; it also negatively regulates the innate immunity signaling pathways, which inhibits the production of IFNs and thereby attenuates the host cell antiviral responses [52]. HIV-1 probably employs these MARCH8 traits to maintain a low-level replication in MDMs, evading host immunosurveillance. Indeed, MDMs serve as the latent reservoir in the long-term infection of HIV-1 [67,68], which is hard to eradicate for the host immune system. Therefore, silencing endogenous *March8* expression may help clear HIV-1 MDMs’ latent infection [69]. Similarly, MARCH8 restricted SVCV infection [37]. However, SVCV infection up-regulated z-MARCH8 expression, attenuating the host’s antiviral innate immunity and thus overall facilitating virus infection [60]. Thus, in this type of cases, the viruses no longer need to evolve a viral gene to counteract MARCH8’s antiviral potency. Conversely, MARCH8 acts as a sheltering umbrella for virus infection under these situations, though the viral replication level is lower.

Alternatively, viruses utilize MARCH8 E3 ligase activity to enhance cellular infection. HCV NS2 utilized MARCH8-catalyzed ubiquitination to promote viral envelopment [59], thereby increasing its intracellular infection. Thus, MARCH8 acts as a helper for HCV cellular infection. Apparently, under this pattern, HCV does not evolve to antagonize MARCH8 or the ubiquitination of NS2 by MARCH8 is an evolutionary counteracting mode.

(II) Viruses evolve a viral gene or strategy to combat MARCH8. Unlike HIV-1, SVA 2AB targeted MARCH8 for lysosome degradation [51], attenuating the host antiviral responses and enhancing virus replication. Likewise, IAV H1N1 M2 escaped from MARCH8-induced degradation via its CT K78/K79 variation [35]. These studies demonstrated that under MARCH8-imposed pressure, some viruses needed to utilize a gene or mode to antagonize its antiviral effects. It also reflects an intracellular competition between the viruses and host cells, which could enlighten us to develop novel antiviral strategies.

(III) Whether viruses evolve a strategy to overcome MARCH8’s antiviral restriction is unclear. MARCH8 is highly expressed in MDMs [27,30] (MDMs also expressed high levels of MARCH1 and MARCH2 [30]) and lung tissue cells [12], which are the infection targets of EBOV and SARS-CoV-2, respectively. Both EBOV GP and SARS-CoV-2 S were found to be potently restricted by MARCH8 in pseudovirus infections [10,33,34]. However, whether the EBOV and SARS-CoV-2 bona fide infections are still sensitive to MARCH8 restriction or have evolved counteracting strategies is unclear. As for SARS-CoV-2, as discussed above, MARCH8 was demonstrated to induce its N/M/S degradation [31,34,44] and S cleavage deficiency [33]. Therefore, it would be intriguing to investigate whether a mechanism exists for SARS-CoV-2 to antagonize MARCH8. Notably, a recent work indicated the host SERINC5 targeted SARS-CoV-2 S glycoprotein and inhibited viral replication. In this case, SARS-CoV-2 employed its ORF-7 [70] and encoded small RNAs [71] to counteract SERINC5-imposed restriction, similar to HIV-1 Nef and MLV glycoGag [40,72,73], which should provide some hints to dissect the possible SARS-CoV-2-MARCH8 combating mechanism. MDMs are believed to be the initial targets of EBOV early-stage infection. Therefore, like SARS-CoV-2, whether EBOV needs to evolve a mode to antagonize MARCH8 restriction and thus keep its effective replication in MDMs deserves further exploration.

In summary, we briefly introduce the recent antiviral advances of MARCH8 in this text. Though MARCH8 mainly negatively regulates viral infection and innate immunity signaling, the virus could occasionally hijack this protein to improve cellular infection. Indeed, MARCH8 and some of its family members set a powerful intrinsic immunity barrier to many viral infections via the CTD and CTI antiviral modes. Thus, whether viruses evolve genes or modes to counteract MARCH restriction, as SVA and IAV H1N1 did, should be further explored in future work, which might help develop novel antiviral therapeutics.
